# Impact of COVID-19 on antibiotic prescriptions for Brazilian children

**DOI:** 10.1038/s41598-022-27212-9

**Published:** 2022-12-30

**Authors:** Fernando de Sá Del Fiol, Isaltino Pereira Andrade

**Affiliations:** Doctoral Program in Pharmaceutical Sciences, University of Sorocaba, Rod. Raposo Tavares, km 92,5, Sorocaba, SP Brazil

**Keywords:** Microbiology, Medical research

## Abstract

The COVID-19 brought a new model of sanitary behavior (social distancing, etc.) that may have reduced the transmissibility of respiratory diseases, reflecting in the number of antibiotic prescriptions. This study evaluated antibiotic prescriptions for children throughout Brazil, between 2014 and 2021. An interrupted time series was conducted to assess variations in antibiotic consumption by the 1–12-year-old children. Joinpoint regression was used to assess the monthly variations. 86 million prescriptions were evaluated. On average, there was a 54% reduction in prescriptions during the pandemic. Amoxicillin showed a reduction of 65%. Joinpoint regression showed that the pandemic altered the prescription growth curve, changing the trend to a 22% drop per month. The new sanitary behavior showed us that it is possible, decreasing the use of antibiotics, to control the children’s respiratory health.

## Introduction

The coronavirus (COVID-19) pandemic has caused numerous worldwide economic, social, and health repercussions. Social distancing, face masks, and children’s classes in an “online” form have brought a notable decrease in medical consultations motivated by respiratory infections^1,2^, affecting the prescription of antibiotics, especially for children. The decrease in physical contact between children brought a decrease in the transmission of respiratory infectious diseases, a decrease in medical consultations and certainly, a decrease in antibiotic prescriptions in this age group during the pandemic period In Norway, the drop in antibiotic prescriptions for children aged 0–4 years was 73% when compared to 2019^3^. In Canada, the reduction occurred in the same proportion (72%) for this age group^4^. In Brazil, until 2010 there was no need to present a medical prescription for the purchase of antibiotics, so that they were considered, in fact, over-the-counter drugs, showing annual increases in consumption^5^. With the obligation of the new legislation, sales in pharmacies began to occur only under medical prescription and, as a result, sales fell, especially macrolides and penicillins^6^. Despite these data showing decreases in consumption, there is no data in the literature showing trends in antibiotic consumption in children in Brazil, before or even during the pandemic, precisely because of the lack of control by national health agencies. Due to this lack of data on the consumption of antibiotics in Brazilian children, we evaluated the number of antibiotic medical prescriptions for children in Brazil between 2014 and 2021 to examine a possible change in the consumption of antibiotics that the pandemic may have brought^7^.

## Methods

An interrupted time series was conducted to assess variations in antibiotic consumption by the 1–12-year-old children between 2014 and 2021 (July). The data were obtained from The National Controlled Products Management System (SNGPC) that contains data on the number of antibiotic units sold in pharmacies throughout Brazil. Data selection only considered medical, not dental or veterinary, prescriptions. Joinpoint regression was used to assess the monthly variations. Statistics was applied to identify the best-fitting points to determine statistically significant changes in time-series data^8^. To compare the average monthly sales between 2014 and the pandemic period, analysis of variance (ANOVA), followed by the Dunnett multiple comparison test (Graph Pad Instat, version 3.05), was performed.

### Ethics approval and consent to participate

The present study used the Brazilian government's medicine use database, so the approval of an ethics committee was not necessary.

## Results

During 2014 (Jan) and 2021 (July), 76,895,784 prescriptions involving antibiotics for 1–12-year-old children were evaluated. Amoxicillin (with or without beta-lactamase inhibitors) represented 55% of prescriptions, followed by azithromycin (18.8%), cephalexin (12.3%), SXT (sulfamethoxazole and trimethoprim) (5.3%), and ceftriaxone (4.1%). In addition, we found metronidazole, cefadroxil, levofloxacin, benzylpenicillin-benzathine, and erythromycin in less than 2% of the prescriptions.

**Figure 1:**
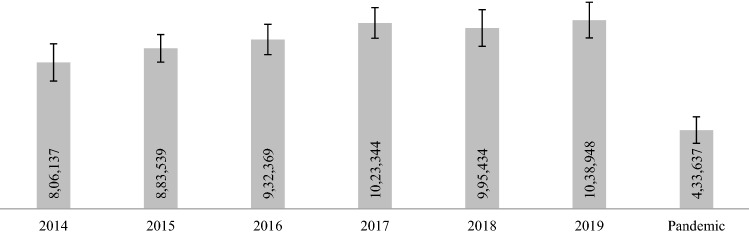
Average monthly consumption (± standard deviation) of antibiotics in Brazil, by children aged 1–12 years, during 2014–2019 and the pandemic period (Jan 2020–July 2021).

The data in Fig. 1 reveal a significant reduction in the average monthly consumption of antibiotics during the pandemic (*P* < 0.0001). Historical data show average sales of around 800,000 units/month in 2014, reaching more than 1 million/month in 2019. However, sales dropped significantly during the Pandemic period (Jan 2020–July 2021), showing a reduction in the monthly average of about 54% in sales (*P* < 0.0001), reaching 433 thousand units/month.

The data in Table 1 show the monthly averages of units sold for all antibiotics between 2014 and 2019 and during the pandemic period and the variation in sales (%). When comparing the sales of all antibiotics during the pandemic with previous years’ averages, they appear to have dropped by 54%. For the most prescribed antibiotic in the country, amoxicillin, the reduction in sales was 65%.

**Table 1:** Monthly average of commercial units of antibiotics sold in Brazil for children aged 1–12 years, between 2014 and 2019 and during the pandemic period (Jan 2020 to July 2021).

	2014	2015	2016	2017	2018	2019	Pandemic	Sales Reduction (%)
Amoxicillin	460,879	485,696	514,597	586,303	576,264	603,683	186,655	65.3*
Azithromycin	137,195	149,734	165,952	186,835	188,782	198,973	110,989	35.1*
Cefalexin	101,731	108,492	119,554	116,504	111,688	112,854	73,262	34.4*
SXT	49,747	53,221	53,180	50,007	45,185	45,841	26,990	45.5*
Ceftriaxone	46,871	40,391	31,520	40,286	40,894	43,614	15,667	61.4*
Cefadroxil	13,542	12,943	12,330	11,870	11,063	10,947	5403	55.4*
Metronidazole	20,898	19,611	19,986	17,668	11,313	12,818	8834	48.1*
Levofloxacin	4,414	5,179	6,306	7,249	7376	6750	3990	35.7*
Penicillin G benzathine	2,445	2,908	5,088	3,836	2,644	3460	1847	45.6*
Erythromycin	6,823	5,364	3,856	2,786	225	8	1	99.9*
Total	806,137	883,539	932,369	1,023,344	995,434	1,038,948	433,637	54.1*

*Statistically significant reduction – (*P* < 0.0001). Dunnett Multiple Comparisons Test. SXT – (sulfamethoxazole and trimethoprim).

**Figure 2:**
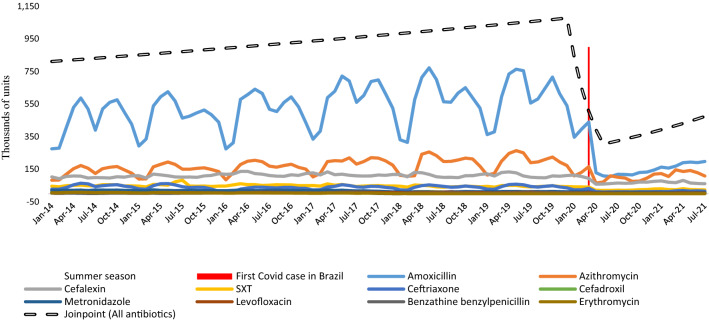
Monthly average of antibiotic sales (2014 Jan–2021 July), beginning of the pandemic in Brazil (red line), summer season (blue bars), and Joinpoint regression (dashed line).

The data in Fig. 2 show the monthly variation in the consumption of the 10 antibiotics most sold in Brazil for children. The blue bars mark the hottest months of the year (summer), making the annual seasonality clear in antibiotic prescriptions for the treatment of respiratory infections (amoxicillin and azithromycin). The figure also marks (in red) the date of the first COVID case in Brazil, coinciding with the abrupt drop in the sale of all antibiotics intended for children. The evaluation using Joinpoint regression (dashed black line) shows, from January 2014, an average monthly growth of 0.4% in the sales of all antibiotics until January 2020, when it marks an average monthly decrease in sales of around 22.3%. The average monthly drop continued until May 2020.

## Discussion

We found a significant reduction in the consumption of antibiotics by Brazilian children during the pandemic. Although some studies in adults^9,10^ have shown an increase in consumption of azithromycin during the pandemic due to its alleged activity against COVID, these same studies also showed a reduction in the consumption of azithromycin in children of approximately 35% during the study period.

Historical data also show a steady drop (regardless of the pandemic) in erythromycin prescriptions. With an average of 6800 monthly prescriptions in 2014, and practically zero number of units was sold in 2019 and during the pandemic. Certainly, the dosage convenience of azithromycin has overcome the discomfort of 4 daily doses of erythromycin, leading physicians to prescribe azithromycin instead^11–13^.

The greatest decreases found in the present study were for amoxicillin (65%) and cephalexin (61%), antibiotics used for respiratory infections in children. This data can also show us that there is a concrete possibility to reduce the consumption of antibiotics in normal situations (without a pandemic). As other similar studies have shown, these data show us that health and educational measures can reduce the need for medical consultations and the consequent prescription of antibiotics^3,3^. In June 2021, with the decrease in cases of covid in Brazil, decrease in social distancing, and winter, average sales began to grow approximately 3.21% per month, continuing until the end date of the study (July 2021).

As other authors have shown^14–16^, social distancing and the new sanitary habits imposed by the pandemic (masks and hand hygiene) are fundamental for reducing respiratory infections and the consequent use of antibiotics. Although the pandemic has brought countless personal and humanitarian tragedies, it has shown that the new norms of sanitary behavior can be fundamental in reducing the transmissibility of respiratory infectious diseases and the consequent decrease in the consumption of antibiotics. Furthermore, a global decrease in prescriptions and consumption of antibiotics will likely decrease the emergence of new resistant bacteria. Consequently, the likelihood of a new, this time of antibiotic-resistant bacteria, pandemic may decrease.

Our results showed that there was a 54% reduction in the consumption of all classes of antibiotics by children during the pandemic. Hygiene measures, social distancing and the reduction in medical appointments may have contributed to this reduction. Other studies will be carried out with updated data to observe the variations in the number of prescriptions of these same antibiotics after the pandemic period.

### Study limitations

The data from the present study, although very representative, only show the antibiotics consumed by Brazilian children in the community (oral and intramuscular). Data on antibiotic consumption in hospitalized children are not included in the present study as they are not available.

## Data Availability

The dataset analysed during the current study is public and available from the corresponding author on reasonable request or at the link: https://dados.anvisa.gov.br/dados/SNGPC/Industrializados/.
